# Exploring Tourism Efficiency and Its Drivers to Understand the Backwardness of the Tourism Industry in Gansu, China

**DOI:** 10.3390/ijerph191811574

**Published:** 2022-09-14

**Authors:** Dan Xue, Xianzong Li, Fayyaz Ahmad, Nabila Abid, Zulqarnain Mushtaq

**Affiliations:** 1 School of Business, Changzhou University, Changzhou 213164, China; 2 Institute of Urban and Rural Civilization, Changzhou University, Changzhou 213164, China; 3 School of Economics, Lanzhou University, Lanzhou 730000, China; 4 Department of Economia Aziendale, University of Gabriele D’Annunzio Cheiti-Pescara, 65127 Pescara, Italy; 5 School of Economics and Finance, Xi’an Jiaotong University, Xi’an 710049, China

**Keywords:** tourism efficiency, Super-SBM, Tobit model, influencing factors, Gansu Province

## Abstract

Gansu Province is rich in tourism resources, and it is the hometown of the “copper galloping horse”, which is the logo of China’s tourism. However, the scale and revenues of tourism in Gansu province are still at a low level. This paper first evaluated the tourism efficiency of 14 cities and prefectures of Gansu Province in China from 2011 to 2019 using the super-slack-based measure (Super-SBM) and then investigated the internal driving mechanism of the efficiency change through the Global Malmquist-Luenberger (GML) index and its decomposition, and finally analyzed the external influencing elements of tourist efficiency by the Tobit model. The results revealed that the tourism efficiency of Gansu Province had increased rapidly during the study period, especially after 2016, the rising range increased. From 2011 to 2019, the cumulative changes in GML index, technological change (TC), and efficiency change (EC) of tourism efficiency in Gansu Province were 5.053, 4.145 and 1.160, respectively, indicating that the improvement of tourism efficiency in Gansu province is mainly due to technological progress. The regression results of the Tobit model show that the status of the tourism industry, trade openness, information level, and technological innovation level can significantly promote tourism efficiency in the province. At the same time, upgrading the industrial structure and the improvement of greening coverage inhibit tourism efficiency. However, the impact of the economic development level on the tourism efficiency of Gansu Province is not apparent. According to the research results, this paper puts forward corresponding suggestions to promote the development of tourism in Gansu Province. This study is crucial for hospitality, tourism, and policy sectors to understand the underlying factors and promote the healthy development of the tourism industry in Gansu Province.

## 1. Introduction

In China, the tourism sector has gained a reputation as a critical industry indulging in regional economic growth and structural reform and improving individual living standards [1]. The total tourism scale of China has expanded rapidly, and the tourism economy has thrived in the past two decades. In 2019, China received 6.15 billion domestic and foreign tourists, accounting for 10.31% of the national tourism employment. Tourism is the industry of “discovering beauty, creating happiness, and sharing happiness”, which is essential to improve people’s happiness and meet people’s pursuit of a better life [2,3]. Meanwhile, tourism is an industry with strong relevance, which can significantly drive and promote service-related industries’ development through the tourism multiplier effect and play a significant role in fostering regional economic development [4].

Gansu Province is rich in tourism resources, and it is the hometown of the “copper galloping horse”, which is the logo of China’s tourism. Gansu Province has seven world cultural heritages, and many tourism resources are unique, monopolistic, and irreplaceable. However, the scale and revenues of tourism in Gansu Province are still at a low level. The tourism products are single, and lack personality, the tourism supporting services level is not high, and the invalid tourism supply does not match the people’s growing demand for high-quality tourism. It is a “province with large tourism resources and small tourism industry”. In 2019, the total tourism revenue of Gansu Province was CNY 268 billion, accounting for only 1.15% of the whole country, which is equivalent to 21.75% of Guizhou Province, 23.12% of Sichuan Province, 24.29% of Yunnan Province and 37.16% of Shaanxi Province, which also belong to the western region of China. There is a need to realize that Gansu Province has backward economic development in western China. It is not suitable for large-scale industrial development due to drought, lack of rain, and a fragile ecological environment [5]. For Gansu Province, it is more necessary to make good use of rich tourism resources, improve the development of tourism, and use tourism development to drive the high-quality development of the economy [6]. Therefore, it is a need to study the tourism efficiency of Gansu Province. This paper aims to make an objective evaluation of the current situation of tourism efficiency in Gansu Province, analyze its internal and external driving factors of tourism efficiency, and put forward effective policy measures to promote tourism efficiency in Gansu Province. It is expected to provide a reference for Gansu Province to improve the quality of tourism industry development.

The innovations of this paper are as follows: firstly, this paper selected Gansu Province as the research object, which is rich in tourism resources but relatively backward in economic development. The tourism products in Gansu Province are single and lack individuality, the service function of tourism is not high, and the scale and revenues of tourism are at a low level, which is typical and representative in Western China. Secondly, by employing the GML index and Tobit model, this paper investigates the influencing factors of tourism efficiency in Gansu Province from both internal and external factors, which makes a methodological contribution to the tourism literature. Thirdly, when measuring tourism efficiency, many scholars use the sum of the number of A-level scenic spots as the input indicators of tourism resources attraction, while this paper gives corresponding scores to different A-level scenic spots and uses the finally calculated scores to represent the tourism resource attraction index. The index selection of tourism efficiency is more scientific.

Section 2 gives an overview of the literature, followed by Section 3 elaborating on the study area. Section 4 discusses the model in detail. Section 5 presented results and interpretation. Lastly, Section 6 concludes the conclusions of this paper and puts forward suggestions according to the research results.

## 2. Literature Review

### 2.1. The Measurement of Tourism Efficiency

Tourism efficiency is an essential means for measuring the comprehensive utilization of tourism resources and evaluating the development level of the tourism industry [7]. Academic research on Tourism efficiency began to focus on tourism hotels [8,9], travel agencies [10], and tourism transportation [11], and then extended to the study of tourism efficiency in the general sense [12,13]. For instance, Corne [14] calculated the efficiency of French tourist hotels and considered that budget hotels have higher efficiency than other types. Köksal and Aksu [15] evaluated the relative operational efficiency of 24 Group A travel agencies operating internationally in Turkey.

Recently, many scholars have studied China’s tourism efficiency and obtained abundant achievements. The study sites included the whole country [16], China’s coastal cities [17], the “Belt and Road” area [18], the Yangtze River delta [19], and the forest park [20]. The research methods mainly included the stochastic frontier method (SFA), data envelopment analysis (DEA), and the Malmquist–Luenberger index (ML). For instance, Peng et al. [21] studied the evolution and characteristics of tourism eco-efficiency in China’s Huangshan National Park. Li and Liu [22] revealed the provincial tourism efficiency of China using the three-stage DEA method. Chaabouni [7] applied a two-stage double bootstrap DEA method to calculate the tourism efficiency of China and considered that China’s tourism efficiency was low. Yang et al. [23] evaluated China’s tourism poverty alleviation efficiency by two stages of the DEA method and Malmquist index and analyzed them by classification.

However, we can see that most of the existing research focused on the tourism efficiency of the whole country, the “Belt and Road”, a certain type of tourism resource area, and economic belts, while less research on the tourism efficiency of specific provinces. The studies on the tourism efficiency of Gansu Province are even rarer. Scholars consider that there is a large gap in tourism efficiency in China [24]. The level of tourism efficiency in Western China is lower than that in central and eastern China, while that in Gansu is at a lower level [25]. Gansu Province is located in an underdeveloped area of Western China. It is rich in tourism resources, but the ineffective supply of tourism products with a single personality and low service function grade does not match the growing demand for high-quality tourism, which is typical and representative in China’s western region. In this paper, we take Gansu Province as the research object, measure tourism efficiency and form a comprehensive understanding of its regional characteristics in Gansu Province.

### 2.2. The Influencing Factors of Tourism Efficiency

The influencing factors of tourism efficiency have also attracted much attention. Scholars have analyzed the impact of economic development level [26], tourism industrial structure, agglomeration of the tourism industry [27], greening coverage [17], opening up [28,29], and information technology [30] on Tourism efficiency. Li et al. [31] considered that there is a positive relationship between tourism and economical, but not all, circumstances. Li and Liu [22] believed that the tourism industry agglomeration significantly impacts tourism efficiency. Tang [32] analyzed the internal relationship between Japan’s trade facilitation and inbound tourism efficiency and believed that trade facilitation promoted Japan’s inbound tourism efficiency. Chaabouni [7] considered that temperature, trade openness, and the number of hotels increased the tourism efficiency of China. Endo [29] argued that the role of foreign direct investment (FDI) in tourism was much more critical in developing countries. Wang et al. [30] believed that technology is the main driving power of tourism eco-efficiency in China, and social civilization level and traffic conditions are the core external factors to promote the tourism eco-efficiency in China. Cao et al. [33] believed that economic development, resource endowment, industrial structure, transportation development, information technology, and institutional supply affect tourism efficiency. Choi et al. [34] found that pure technology inefficiency led to festival tourism’s inefficiency.

The research on the influencing factors of tourism efficiency has achieved rich results. However, we found that there are few studies on the influencing factors of tourism efficiency in Gansu Province. We need to note that the influence factors of tourism efficiency are different between different regions. The existing research on the influencing factors of tourism efficiency in the whole country and the developed areas of the eastern region has certain regional limitations. As Gansu Province, which is located in Northwest China, has abundant tourism resources and underdeveloped economic development, the factors affecting its tourism efficiency may be different from the factors affecting the whole country and other developed regions and provinces. Additionally, most of the existing studies use the regression method to study the influencing factors of tourism efficiency, and there is still a lack of research on the influencing factors of tourism efficiency both from internal driving and external influencing factors. By using the GML index and Tobit model, this paper studies the influencing factors of tourism efficiency from both internal and external factors.

## 3. Study Area

**Figure 1:**
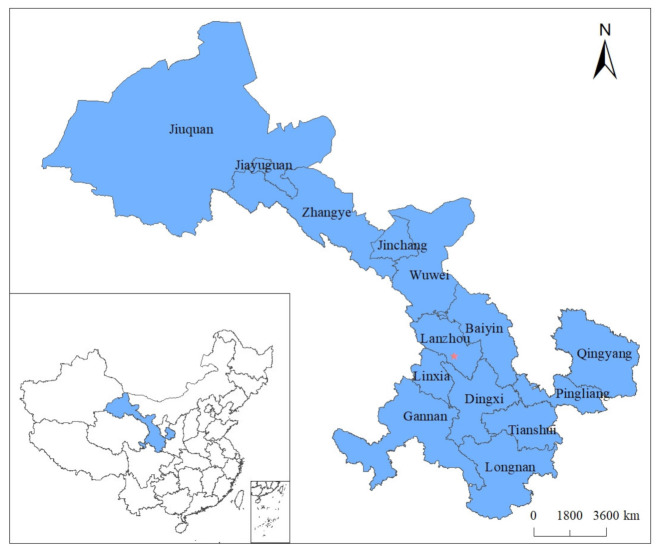
Study area.

The study area of this paper is the Gansu Province of China. Gansu Province is rich in tourism resources. Many resources are unique and irreplaceable in the country and even in the world. By the end of December 2019, Gansu Province had 6 5A scenic spots, 99 4A scenic spots, and 132 3A scenic spots, receiving 370 million tourists and realizing tourism revenue of CNY 268 billion. The study area of this paper is shown in Figure 1.

## 4. Methodology

This paper used the panel data of 14 cities and prefectures in Gansu Province from 2011 to 2019, evaluated the tourism efficiency with the Super-SBM model, analyzed the temporal change and spatial characteristics of tourism efficiency in different cities in Gansu Province, used the GML index to explore the internal driving factors of the tourism efficiency change, and analyzed the external influencing factors of tourism efficiency in Gansu Province through Tobit model.

### 4.1. Research Models

#### 4.1.1. The Super-SBM Model

Among the current methods to measure tourism efficiency, the most widely used method was the data envelopment analysis (DEA). DEA method is a method that uses linear programming theory to study the effectiveness of input and output systems in public programs, which was proposed by Charnes et al. in 1978 [35]. Most traditional DEA models are radial or angular and do not reflect the relaxation of the input or output index. Tone [36] put forward a non-radial and non-angular SBM model, which considered the proportional improvement between the current state of the invalid decision-making unit and the strong target value and considered the relaxation improvement. The SBM model overcomes some limitations of the traditional DEA model, but there were also some shortcomings. When the efficiency value is greater than 1, it cannot distinguish the effective decision-making units (DMUs). Tone [37] also proposed the Super-SBM model, which allowed the efficiency value to be greater than or equal to 1, further distinguishes the effective DMUs, and avoids the problem that the effective DMUs cannot be compared.

Suppose there are *n* tourism production DMUs; each DMU has *m* kinds of input elements and $s_{1}$ kinds of desirable output elements, $s_{2}$ kinds of undesirable output elements. $x\in R_{m}^{}$ $x\in R_{m}^{}$, $y^{g}\in R_{s_{1}}^{}$, and $y^{b}\in R_{s_{2}}^{}$. The matrix forms are X, $Y^{g}$, and $Y^{b}$, respectively, while $X=[x_{1},\cdots ,x_{n}]\in R_{m\times n}^{}$, $Y^{g}=[y_{1}^{g},\cdots ,y_{n}^{g}]\in R_{s_{1}\times n}^{}$, and $Y^{b}=[y_{1}^{b},\cdots ,y_{n}^{b}]\in R_{s_{2}\times n}^{}$. Therefore, the productive possibility sets that do not include DMUs $\left(x_{0},y_{0}^{g},y_{0}^{b}\right)$ can be described as follows:

(1) $$p\backslash (x_{0},y_{0}^{g},y_{0}^{b})=\left\{(\bar{x},{\bar{y}}^{g},{\bar{y}}^{b})|\bar{x}\geq \sum_{j=1}^{n}\lambda_{j}x_{j},{\bar{y}}^{g}\leq \sum_{j=1}^{n}\lambda_{j}y_{j}^{g},{\bar{y}}^{b}\geq \sum_{j=1}^{n}\lambda_{j}y_{j}^{b},\lambda \geq 0\right\}$$

The formula of the Super-SBM model is as follows:

(2) $$\rho^{*}=\min\frac{\frac{1}{m}\sum_{i=1}^{m}\frac{{\bar{x}}_{i}}{x_{i0}}}{\frac{1}{s_{1}+s_{2}}\left(\sum_{r=1}^{s_{1}}\frac{{\bar{y}}_{r}^{g}}{y_{r0}^{g}}+\sum_{u=1}^{s_{2}}\frac{{\bar{y}}_{l}^{b}}{y_{l0}^{b}}\right)}$$

(3) $$s.t.\left\{\begin{array}{c} \bar{x}\geq \sum_{j=1,\neq 0}^{n}\lambda_{j}x_{j},\;j=1,\cdots ,m\\ {\bar{y}}^{g}\leq \sum_{j=1,\neq 0}^{n}\lambda_{j}y_{j}^{g},\;r=1,\cdots ,s_{1}\\ {\bar{y}}^{b}\geq \sum_{j=1,\neq 0}^{n}\lambda_{j}y_{j}^{b},\;l=1,\cdots ,s_{2}\\ \bar{x}\geq x_{0},{\bar{y}}^{g}\leq y_{0}^{g},{\bar{y}}^{b}\geq y_{0}^{b},\lambda \geq 0,\sum_{j=1,\neq 0}^{n}\lambda_{j}=1 \end{array}\right.$$

where $\rho^{*}$ represents the efficiency of the Super-SBM model, *x* is the input vector, *y* is the output vector, λ is the weight. $s^{-}$ is the slack variables for the input vector, while $s^{g}$ is the slack variables for the output vector. The “-” above the variable represents the projection value, and the subscript 0 in the lower right corner of the variable represents the evaluated DMUs. $\rho^{*}$ is greater than 0, the bigger the $\rho^{*}$ value, the higher the efficiency level. In the above formula, when the slack variables are equal to 0, $\rho^{*}$ are more significant than or equal to 1, indicating that the DMUs are efficient. When it is less than 1, it suggests that the DMUs have efficiency loss.

#### 4.1.2. The Global Malmquist-Luenberger Index (GML)

Malmquist index can dynamically reflect the change and trend of the research object’s relative efficiency. The traditional Malmquist index cannot be compared across periods. In contrast, the GML index took the sum of each period as the reference set, which can be compared across periods when studying efficiency [38]. In this paper, according to the research of Pastor and Lovell [39], the GML index based on the Super-SBM distance function from period *t* to period *t* + 1 was defined as follows:

(4) $$\begin{array}{l} GML_{t}^{t+1}(x^{t},y^{t},b^{t},x^{t+1},y^{t+1},b^{t+1})\\ =\frac{E^{g}(x^{t+1},y^{t+1},b^{t+1})}{E^{g}(x^{t},y^{t},b^{t})}\\ =\frac{E^{t}(x^{t+1},y^{t+1},b^{t+1})}{E^{t}(x^{t},y^{t},b^{t})}\times \left[\frac{E^{g}(x^{t+1},y^{t+1},b^{t+1})}{E^{t+1}(x^{t+1},y^{t+1},b^{t+1})}\times \frac{E^{t}(x^{t},y^{t},b^{t})}{E^{g}(x^{t},y^{t},b^{t})}\right]\\ =EC_{t}^{t+1}\times TC_{t}^{t+1} \end{array}$$

In the above formula, the reference set is the collection of each period $p^{g}(x)=p^{1}(x^{1})\cup p^{2}(x^{2})\cup p^{3}(x^{3}),\cdots ,p^{t}(x^{t})$. $E^{g}(x^{t},y^{t},b^{t})$ is the global distance function, which is solved by the Super-SBM model. If the $GML_{t}^{t+1}$ index is less than 1, equal to 1, or greater than 1, it means that the tourism efficiency decreases, remains unchanged, or increases from period *t* to period *t* + 1, respectively. The GML index can be decomposed into the multiplication of the EC index and the TC index. $EC_{t}^{t+1}$ represents the change of the maximum impending degree of the actual output and production frontier of tourism in Gansu Province from period *t* to period *t* + 1. $TC_{t}^{t+1}$ illustrates the movement of the production frontier of tourism in Gansu Province from period *t* to period *t* + 1. The value of $EC_{t}^{t+1}$ and $TC_{t}^{t+1}$ greater than 1 (less than 1) represents the improvement (decline) of efficiency and technological progress (retrogression) from period *t* to period *t* + 1, respectively.

#### 4.1.3. The Tobit Model

The tourism efficiency of 14 cities in Gansu Province is greater than 0, which belongs to the “limited dependent variables”, the ordinary least squares (OLS) regression may cause bias and inconsistency [40]. Nevertheless, the Tobit model can solve the problem of “limited dependent variables” [41]. Consequently, this paper established the Tobit model to evaluate the influencing factors of tourism efficiency of Gansu Province and explore the size and direction of each influencing factor. The expression of the Tobit model is as follows:

(5) $$Y_{it}=\left\{\begin{array}{l} Y_{it}^{*}=\beta_{0}+\sum_{t=1}^{n}\beta_{t}x_{it}+\mu_{i},Y_{it}^{*}>0\\ 0,Y_{it}^{*}\leq 0 \end{array}\right.$$

where $Y_{it}$ is the explained variable, representing the tourism efficiency of the DMU *i*. $x_{it}$ is a series of the explanatory variable, and the selection of each specific explanatory variable will be introduced in Section 4.2.2. $\beta_{0}$ is the constant term; $\beta_{t}$ is the estimation coefficient, t=1,2,…,14 is a unique random error disturbance term; $\mu_{i}$ is the single random error perturbation term, and $\mu_{i}\sim (0,\sigma^{2})$.

### 4.2. Data and Indicators

This study assessed the tourism efficiency and driving factors of 14 cities and prefectures in Gansu Province from 2011 to 2019. Among the indexes of the SBM model, the list of A-level scenic spots, the numbers of travel agencies, and star-rated hotels were calculated from the website of the Gansu Provincial Tourism Development Commission. The indicator of numbers of tertiary industry employees was obtained from the “Gansu Development Yearbook”. The indicators of total tourism revenue and total tourist arrivals were collected from the statistical bulletin of cities and states in Gansu Province each year. Among the variables of the Tobit regression model, the tourism efficiency was calculated in Section 5.1, and the remaining indicators were derived from the “Gansu Development Yearbook”, “China City Statistical Yearbook”, and “China Urban Unified Construction Design Yearbook”.

#### 4.2.1. Input-Output Indicators for Super-SBM Model and GML Index

The indicators to measure tourism efficiency with Super-SBM Model include input indicators and output indicators. The tourism resource attraction is an essential input factor of tourism production. Some scholars used the number of scenic spots above Grade A or 3A to reflect the overall attraction of tourism resources. Still, the attraction of scenic spots at different levels is quite different. Only adding up the number of scenic spots above Grade A or 3A may ignore the fact that scenic spots of different levels have different attractions. Based on the detailed rules of the “Landscape Quality Evaluation Rules” (Detailed Rules 2) in the national standard assessment rules of “Quality Rating and Classification of Tourist Attractions” formulated by the National Tourism Administration of China, this paper assigned 90 points for 5A-level scenic spots, 85 points for 4A-level, 75 points for 3A-level, 60 points for 2A-level, and 50 points for A-level. The tourist attraction scores of 14 cities in Gansu Province were calculated as an essential input factor of tourism efficiency.

The labor force is another input factor of tourism efficiency. The number of tourism direct employees is the ideal index, but the relevant statistics do not sort out the data of tourism direct employees in the city and prefecture level of Gansu Province. Owing to data availability, this paper chose the number of tertiary industry employees to replace it. Additionally, travel agencies are the intermediary and bridge of tourism development, and star hotels are an important guarantee for tourists’ accommodation. Referring to the research of Ji et al. [42], this study took travel agencies and starred hotels as the other two input factors of tourism efficiency.

This paper selected tourism economic output and tourism scale output to measure the output indicators of tourism efficiency in Gansu Province according to the research of Li et al. [22]. Among them, tourism economic output was expressed by tourism revenue, and tourist arrivals expressed tourism scale output.

The index system and descriptive statistics of input-output indicators of tourism efficiency of Gansu Province during 2011–2019 are displayed in Table 1.

In this paper, the variables for the GML index are the same as those of the Super-SBM Model. The GML index and its decomposition of tourism efficiency in Gansu Province were calculated based on the distance function of Super-SBM through Formula (4).

#### 4.2.2. Variable Selection for the Tobit Model

This paper selected the tourism efficiency of 14 cities in Gansu Province during 2011–2019 as the explained variable, which will be calculated in Section 5.1. According to previous research and the actual situation of Gansu Province, this paper selected the economic development level (lnPGDP), industrial structure (IS), tourism industry status (TIS), trade openness (TO), information level (IN), urban greening level (UG) and technical innovation (TI) as the explanatory variables of the Tobit model. Specifically, the present study selected the most commonly used per capita GDP to express the economic development level and convert it to the constant price of 2011. We also took the logarithm of it to eliminate the impact of heteroscedasticity. According to the research of Liang et al. [43], this paper selected the ratio of tertiary industries to secondary industries to express the industrial structure. Based on the study of Qiu et al. [17], we selected the proportion of total tourism revenue to GDP to represent the tourism industry status. Drawing on the research of Chaabouni [7], this paper selected the ratio of the total import and export trade in GDP to measure trade openness. The information level was expressed by the total value of post and telecommunications business; we took the logarithm of the variables in order to avoid multicollinearity. In this paper, the urban greening level was expressed by the green coverage rate of the built-up area, and technical innovation was measured by the research and development (R&D) expenditure [44].

## 5. Results and Discussion

### 5.1. The Results and Discussion of the Tourism Efficiency in Gansu Province

#### 5.1.1. The Temporal Evolution of Tourism Efficiency in Gansu Province

**Figure 2:**
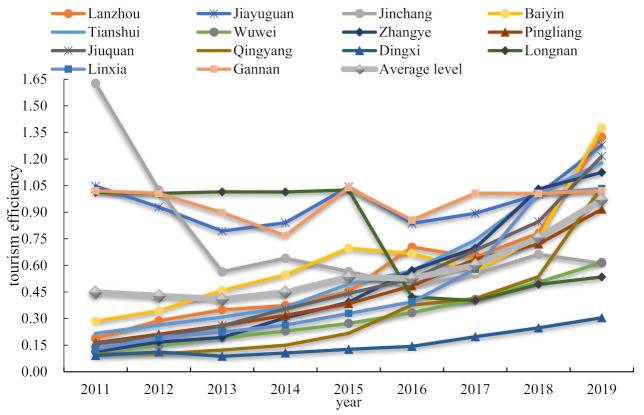
The changing trend of tourism efficiency in Gansu Province during 2011–2019.

The evolution characteristics of tourism efficiency of 14 cities in Gansu Province during 2011–2019 are shown in Figure 2. We can see that the average level of tourism efficiency in Gansu Province showed a fluctuating upward trend during 2011–2019. From 2011 to 2014, the average efficiency curve showed a horizontal state, and the efficiency level changed little. It rose slowly from 2014 to 2016, and the rising speed was significantly accelerated after 2016. This finding is similar to the research of Peng and Chen [45]. During the “China’s 13th Five-Year Plan” period, Gansu Province attached great importance to tourism development, proposed vigorously developing global tourism, and emphasized the integration of tourism and other industries. Therefore, after 2016, the tourism efficiency of Gansu Province has dramatically improved.

From the perspective of cities in Gansu Province, the tourism efficiency level of Jiayuguan City and Gannan Tibetan Autonomous Prefecture maintained a high level each year during the study time frame. Jiayuguan City is a famous historical site, and the scenic spot belongs to the world’s cultural heritage. At the same time, Gannan Prefecture is rich in tourism resources and beautiful scenery. The tourism efficiency of these two areas was maintained at a comparatively high level during the study period. The tourism efficiency of Lanzhou City, Zhangye City, Baiyin City and Jiuquan City has experienced rapid growth from 2011 to 2019. The tourism efficiency values of these three cities were below 0.2 in 2011 and above 1.1 in 2019.

However, the tourism efficiency values of Jinchang City and Longnan City have shown a downward trend. Jinchang City is a typical old-brand mineral resource-based city with few tourism resources; while Longnan City’s transportation infrastructure is relatively lagging, and the accessibility of tourist attractions is low, thence the tourism efficiency of these two cities has declined.

#### 5.1.2. The Spatial Distribution of the Tourism Efficiency in Gansu Province

**Figure 3:**
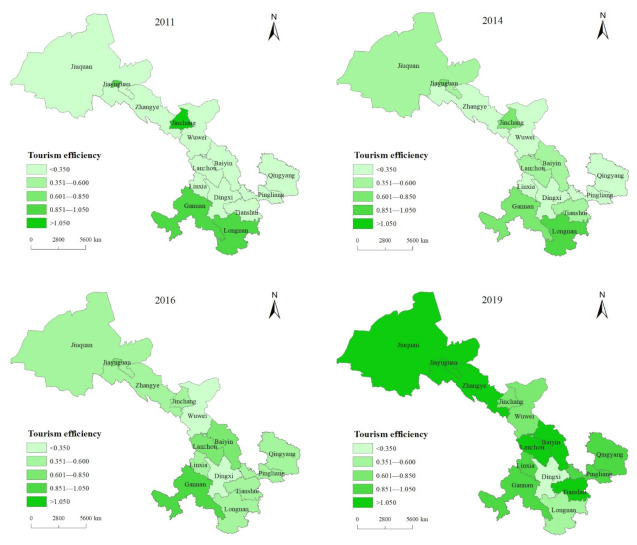
The spatial distribution of tourism efficiency in Gansu Province in 2011, 2014, 2016, and 2019.

Most studies believed that China’s tourism efficiency has obvious regional differences [46], and so does the tourism efficiency within Gansu Province. In order to display the spatial distribution of tourism efficiency in Gansu Province more intuitively, this paper further introduced the tourism efficiency of each city in Gansu Province in 2011, 2014, 2016, and 2019 into ArcGIS10.4 software for visual mapping. The details are presented in Figure 3. In 2011, the tourism efficiency level of Gansu Province was generally low, and the high-efficiency values were relatively scattered. In the distribution map, the efficient colors of Gannan and Longnan City in the south of Gansu Province, Jinchang City in the center of Gansu Province, and Jiayuguan City in the northwest of Gansu Province were darkest, and the colors of other cities were very shallow. In 2014, the efficiency distribution map color was darker than that in 2011, showing the distribution characteristics of “high at both ends and low at the middle”. Longnan City and Gannan Tibetan Autonomous Prefecture in the south of Gansu Province, and Jiuquan City and Jiayuguan City in the north are darker. The colors of tourism efficiency values in central Gansu Province were relatively light, except for Jinchang City. In 2016, the high-efficiency areas in southern Gansu Province moved northward, and the efficiency colors of Lanzhou City and Baiyin City became darker, while the tourism efficiency of Longnan City decreased. In 2019, the color of tourism efficiency’s distribution map had become significantly darker, and the color difference between regions had become smaller. The high-efficiency values were concentrated in Jiayuguan City, Jiuquan City, and Zhangye City in the Hexi Corridor, Lanzhou City, and Baiyin City in the central of Gansu Province, and Qingyang City, Pingliang City, and Tianshui City in southeastern of Gansu Province. Meanwhile, Dingxi City and Longnan City in southern Gansu Province had the lowest tourism efficiency.

### 5.2. The GMLindex and Its Decomposition of Tourism Efficiency in Gansu Province

According to the Super-SBM distance function introduced above, this paper calculated the GML index of the tourism efficiency of 14 cities in Gansu Province during 2011–2019 and decomposed the GML index into EC index and TC index. The details are shown in Table 2.

From 2011 to 2019, the tourism efficiency of Gansu Province increased significantly. The cumulative change value of the GML index was 5.053, and the geometric average value was 1.176. The cumulative change value of the GML index in 12 cities was more significant than or equal to 1, and only two cities’ GML index of tourism efficiency was less than 1. The five cities with the largest cumulative change value of the GML index were Qingyang City (10.285), Zhangye City (9.908), Linxia City (8.162), Jiuquan City (7.705), and Lanzhou City (6.992), indicating that the tourism efficiency of these five cities had increased the fastest within the research time. The two cities with a cumulative change value of GML index of less than 1 were Jinchang City and Longnan City, indicating that the tourism efficiency of these two cities has decreased within the research time.

Judging by the decomposition results of the GML index, the change in tourism efficiency (GML index) in Gansu Province is primarily owing to the technological progress index (TC), which was consistent with the findings of Peng et al. [21]. The cumulative change value and the geometric average value of TC index were 4.145 and 1.166, respectively, while the cumulative change value and the geometric average value of EC index were 1.160 and 1.006, respectively. The TC index of most cities in Gansu Province was more significant than the EC index. Therefore, this article believed that technological progress was the main driving factor in promoting tourism efficiency in Gansu Province.

**Figure 4:**
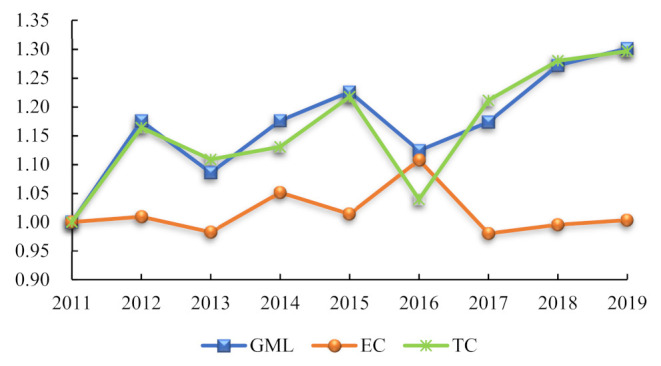
The GML index and its decomposition of tourism efficiency in Gansu Province from 2011 to 2019.

Figure 4 shows the time-series changes of the tourism efficiency GML index and its decomposition results in Gansu Province. It can be seen that from 2011 to 2019, the GML index of tourism efficiency in Gansu Province was more significant than 1, and the GML index showed a fluctuating upward trend. The changing trend of TC was consistent with the changing trend of GML and overlapped at multiple points. The EC index curve was below the GML and TC curves, and the changing trend was precisely the opposite. This also indicated that technological progress had played a decisive role in promoting tourism efficiency in Gansu Province. Local governments in Gansu Province can promote tourism efficiency by improving technological progress.

### 5.3. The Influencing Factors of Tourism Efficiency in Gansu Province

#### 5.3.1. Results of the Tobit Model

This paper made a multicollinearity test on the influencing factors of tourism efficiency in Gansu Province. We found that the maximum value of each variable’s variance inflation factor (VIF) was 2.96, indicating that the degree of collinearity of each influencing factor selected in this paper was within a reasonable range, and there was no need to worry about Multicollinearity. According to the results of the LR test, the Tobit model with random effects was selected for empirical analysis. The regression is shown in Table 3.

The estimated coefficient of economic development level (lnPGDP) did not pass the significance level test, indicating that economic development has no significant impact on tourism efficiency in Gansu Province. A finding was consistent with the research of Pan et al. [47], which studied the tourism efficiency of Shaanxi Province. Generally speaking, the economic development level can provide a sound economic foundation for the tourism industry, and there is a positive relationship between the economic development level and tourism efficiency [31]. However, in Gansu Province, the economic development level is backward, and it has no significant impact on tourism development.

The coefficient of industrial structure (IS) had passed the 1% significance level test; interestingly, the sign was negative, indicating that upgrading of industrial structure will inhibit tourism efficiency in Gansu Province, which is contrary to the research of Lu et al. [27]. The reason may be that, as a resource-based province in Western China, the development of tourism in Gansu Province was still driven by fixed-asset investment, which was closely related to construction and manufacturing. At the same time, the volume of tourism-related industries such as tourism catering and tourism accommodation was small and chaotic in Gansu Province. Therefore, there was a negative correlation between industrial structure and tourism efficiency in Gansu Province.

The coefficient of tourism industry status (TIS) was significantly positive at the 1% level, which meant that improving the degree of tourism specialization would significantly promote tourism efficiency in Gansu Province, which was consistent with the research of Qiu et al. [17]. On the one hand, the status of tourism in the national economy improved, and its contribution to the national economy increased, which will also improve its tourism efficiency. On the other hand, tourism has strong relevance, which can integrate and promote with other industries. The development of other industries can also drive the tourism industry and promote tourism efficiency in Gansu Province. For Gansu Province, improving the status of the tourism industry is an effective means to promote tourism efficiency.

The regression coefficient of trade openness (TO) was positive and passed a 1% significance level test, indicating that trade openness can significantly improve tourism efficiency in Gansu Province, which was consistent with the research of Chaabouni [7]. Tang [32] also got similar results with Japan’s 2011–2019 data. The improvement of trade openness can improve the ability of the local tourism market to absorb external resources, enhance the competitive advantage of the tourism business and improve tourism efficiency. Additionally, trade openness may encourage business travel, promoting tourism development in Gansu Province. Local governments in Gansu Province can improve tourism efficiency by expanding foreign trade and business travel.

The regression coefficient of information level (IN) was significantly positive at the 1% level, which meant that the information level had a positive impact on the tourism efficiency of Gansu Province. The results were consistent with previous research [48]. Information level is the bridge of tourism radiation [49], which promotes interconnection and resource sharing among various regions. Therefore, improving the information level is also an important way to improve tourism efficiency in Gansu Province.

The regression coefficient of urban greening level (UG) was negative and passed the 1% significance level. A finding was just opposite to the research of Qiu et al. [17], which believed that the green coverage rate has significantly promoted tourism efficiency in the eastern coastal areas of China. The reason may be that, contrary to the coastal areas, Gansu Province is dry and rainless, the greening level is low, and the unique desert and Gobi landscape are easier to attract foreign tourists [45,50]. Therefore, there was a reverse relationship between greening coverage and tourism efficiency in Gansu Province.

The regression coefficient of technological innovation (TI) was significantly positive at the 1% level, which meant that improving scientific and technological innovation levels can significantly improve tourism efficiency in Gansu Province. We should pay more attention to technological innovation [51]. The use of advanced technology makes it easier for tourists to participate in tourism activities, which improves the management level of the tourism industry to increase tourism efficiency.

#### 5.3.2. Robustness Test

This study re-estimated the regression results of the Tobit model by replacing variables. In the above regression estimation, the industrial structure index was expressed by the ratio of tertiary industries to secondary industries. At the same time, some scholars also used the ratio of the tertiary industry’s total output value to GDP to characterize it. Additionally, this article used the R&D expenditure to represent the technical innovation level, while some scholars also used the ratio of R&D expenditure to GDP to represent it. In the robustness test, this paper further used the proportion of the tertiary industry’s output value in GDP to replace the industrial structure indicator. It replaced the technical innovation index with the ratio of R&D expenditure to GDP. Other variables remained unchanged, and performed the Tobit regression estimation again. The VIF value of each indicator was within a reasonable range. The results of the robustness test are shown in Table 4. We can see that the regression results of the robustness test were still similar to the previous ones. Overall, we believed that the regression results of the article were robust.

## 6. Conclusions and Policy Implications

Gansu Province is rich in tourism resources, but its scale and revenues of tourism are still at a low level. Measuring the tourism efficiency of Gansu Province and analyzing the reasons for its backwardness are of great importance for the healthy development of tourism in Gansu Province. Using the panel data of 14 cities in Gansu Province from 2011 to 2019, this paper first evaluated the tourism efficiency of Gansu Province with the Super-SBM model, analyzed the temporal and spatial characteristics of tourism efficiency of different cities in Gansu Province, and then explored the internal driving mechanism of the efficiency change with the GML index, and also analyzed the external influencing factors of tourism efficiency with Tobit model. The results were as follows:

Firstly, the tourism efficiency of Gansu Province showed a fluctuating upward trend during 2011–2019. Since 2016, the tourism efficiency of Gansu Province has dramatically improved. The tourism efficiency of Lanzhou City, Zhangye City, and Baiyin Cityand Jiuquan City has increased rapidly, while the tourism efficiency of Jinchang City and Longnan City showed a downward trend. In 2019, the high-efficiency values were concentrated in the cities of Hexi Corridor and the central and southeastern Gansu Province, while the cities of southern Gansu Province had the lowest tourism efficiency, such as Dingxi City and Longnan City. Secondly, the changing trend of the GML index of the tourism industry in Gansu Province is basically consistent with the changing trend of TC, but it is opposite to the changing trend of EC, which means that the improvement of tourism efficiency (GML) in Gansu Province was mainly owing to the technological progress index (TC) change. Additionally, the result of the Tobit model showed that the tourism industry status, trade openness, information level, and technological innovation could significantly promote the tourism efficiency of Gansu Province. Interestingly, we found that upgrading the industrial structure and improving green coverage in Gansu Province will inhibit tourism efficiency. The impact of economic development level on tourism efficiency in Gansu Province was not apparent.

These findings have several policy implications for all stakeholders. For instance, firstly, based on differences in tourism efficiency among cities in Gansu Province, the local government should optimize the allocation of resources, carry out regional tourism cooperation, and strengthen information exchange with surrounding provinces and cities and tourism hot cities to promote tourism efficiency. For this, the provincial government should establish large tourism groups to effectively integrate the tourism resources in the province and improve the management level, and then improve tourism efficiency. Secondly, local governments in Gansu should promote technological innovation and encourage core tourism enterprises to innovate the supply of tourism products. Thirdly, it is necessary to expand tourism revenue and improve the tourism industry’s status in the national economy for improved efficiency. Furthermore, local governments of cities and prefectures in Gansu should also integrate tourism and other industries, seize the opportunities of global tourism to supplement the types of tourism products and improve the comprehensive tourism consumption income. Moreover, the local government of Gansu province can promote smart tourism, make full use of the internet and big data platform, promote the intellectualization of tourism services, and improve the satisfaction of tourists to enhance overall tourism efficiency. Finally, local government should promote openness in terms of foreign trade and encourage business travel in order to improve tourism efficiency. Last but not least, future research can extend this investigation by analyzing the crucial factors to tourism efficiency in other provinces and regions. Therefore, the current study can be used as a base for future projects to draw useful policy measures for China and other tourist destinations worldwide.

## Figures and Tables

**Table 1:** The index evaluation system of tourism efficiency and its descriptive statistics in Gansu Province.

Index	Layer of Indicators	Unit	Obs	Mean	Std. Dev.	Min.	Max.
Input	The attraction of tourism resources	-	126	1232.50	690.43	60.00	3010.00
	Numbers of tertiary industry employees	10 thousand persons	126	10.39	8.47	1.37	48.82
	Numbers of travel agencies	Piece	126	39.90	61.95	5.00	293.00
	Numbers of star-rated hotels	Piece	126	26.70	14.08	5.00	63.00
Output	Total tourism revenue	CNY 100 million	126	88.81	116.77	4.61	765.27
	Total tourist arrivals	10 thousand person times	126	1359.96	1365.45	92.97	8205.02

**Table 2:** The GML index of the tourism efficiency of 14 cities in Gansu Province.

City	Cumulative Change			Geometric Mean		
	GML	EC	TC	GML	EC	TC
Lanzhou	6.992	1.086	6.436	1.275	1.010	1.262
Jiayuguan	1.220	1.245	0.980	1.025	1.028	0.997
Jinchang	0.376	0.451	0.833	0.885	0.905	0.977
Baiyin	4.826	1.390	3.472	1.217	1.042	1.168
Tianshui	5.438	0.855	6.362	1.236	0.981	1.260
Wuwei	5.385	1.115	4.831	1.234	1.014	1.218
Zhangye	9.908	2.222	4.459	1.332	1.105	1.205
Pingliang	5.597	0.917	6.105	1.240	0.989	1.254
Jiuquan	7.705	0.972	7.923	1.291	0.997	1.295
Qingyang	10.285	2.195	4.685	1.338	1.103	1.213
Dingxi	3.321	0.680	4.886	1.162	0.953	1.219
Longnan	0.528	0.415	1.272	0.923	0.896	1.031
Linxia	8.162	1.711	4.770	1.300	1.069	1.216
Gannan	1.000	0.982	1.017	1.000	0.998	1.002
Average level	5.053	1.160	4.145	1.176	1.006	1.166

**Table 3:** Estimated results of the Tobit model.

Variable Name	Coefficient	Std. Error	T-Statistic	*p*-Value	VIF
lnPGDP	−0.008	0.092	−0.09	0.930	2.96
IS	−0.090 ***	0.034	−2.63	0.009	2.91
TIS	0.017 ***	0.002	7.23	0.000	2.25
TO	0.011 ***	0.002	6.55	0.000	1.39
IN	0.144 ***	0.032	4.53	0.000	2.17
UG	−0.012 ***	0.003	−3.57	0.000	2.33
TI	0.109 **	0.049	2.21	0.027	2.17
_cons	−1.079	0.810	−1.33	0.183	
/sigma_u	0.246 ***	0.062	3.96	0.000	
/sigma_e	0.155 ***	0.011	14.41	0.000	
Loglikelihood	34.053		LR test(P)	0.000	
Wald chi2	264.39		N	126	

Note: **and *** are significant at the level of 5% and 1%, respectively.

**Table 4:** Robustness test results.

Variable Name	Coefficient	Std. Error	T-Statistic	*p*-Value	VIF
lnPGDP	0.068	0.073	0.94	0.349	2.62
IS	−0.006 **	0.003	−2.09	0.036	3.58
TIS	0.017 ***	0.002	6.81	0.000	2.03
TO	0.010 ***	0.002	5.33	0.000	1.79
IN	0.146 ***	0.034	4.34	0.000	2.19
UG	−0.014 ***	0.003	−4.28	0.000	2.23
TI	0.035 *	0.020	1.74	0.083	2.78
_cons	−1.627 **	0.652	−2.5	0.013	
/sigma_u	0.162 ***	0.062	3.96	0.000	
/sigma_e	0.166 ***	0.011	14.41	0.000	
Loglikelihood	31.324		LRtest(P)	0.000	
Wald chi2	232.13		N	126	

Note: *, **, and *** are significant at the level of 10%, 5%, and 1%, respectively.
